# Literary Notes

**Published:** 1908-12-05

**Authors:** 


### LITERARY NOTES.

Three books of especial interest to those connected with the tropics are shortly to be published by Messrs. J. and A. Churchill. "Report on the Prevention of Malaria in Mauritius," by Professor Ronald Ross, C.B., F.R.S., includes many illustrations and charts, and is issued by authority of the Crown Agent for the Colonies. A new volume of "Studies from the Institute of Medical Research" is issued by authority of the Government of the Federated Malay States. "Lessons on Elementary Hygiene," by Dr. W. T. Prout, C.M.G., is specially designed for use in tropical climates.

The eighteenth edition of "Squire's Companion to the British Pharmacopœia" is also announced by the same firm. An increase of 500 pages has been necessitated by the issue of new editions of twelve foreign Pharmacopœias. The whole work has been entirely re-written.

